# Analysis of Globular Cells in Corneal Nerve Vortex

**DOI:** 10.3389/fmed.2022.806689

**Published:** 2022-02-22

**Authors:** Ran Hao, Ziyuan Liu, Yilin Chou, Chen Huang, Dalan Jing, Haikun Wang, Shuang Gao, Xuemin Li

**Affiliations:** ^1^Department of Ophthalmology, Peking University Third Hospital, Beijing, China; ^2^Beijing Key Laboratory of Restoration of Damaged Ocular Nerve, Department of Ophthalmology, Peking University Third Hospital, Beijing, China

**Keywords:** corneal nerves vortex, dendritic cells, globular cells, Langerhans cells, *in vivo* confocal microscopy

## Abstract

**Purpose:**

Less was known about globular cells which were a type of dendritic cells (DCs) in cornea. We aimed to investigate the morphological and distribution characteristics of globular cells in corneal vortex and their clinical correlations with ocular surface.

**Methods:**

Case records of patients who underwent *in vivo* confocal microscopy (IVCM) were evaluated retrospectively. The morphology and distribution features of globular cells in cornea nerve vortex and their co-existence status with Langerhans cells (LCs) were analyzed. Data of ocular surface symptoms and signs were collected and their correlations with globular cells distribution patterns and dendritic forms were performed. Dry eye patients without LCs were treated with preservative-free artificial tears, while patients with LCs were treated with artificial tears and fluoromethalone until the activated LCs disappeared.

**Results:**

A total of 836 eyes from 451 individuals were included. Three distribution patterns of globular cells in vortex were investigated, type 1 scattered globular cells (57.66%), type 2 large amounts of globular cells (≥50 cells) gathering in vortex and along some fixed vortex direction horizontally (13.52%) and type 3 no globular cells (28.83%). Their location and cell count altered slightly in the follow-ups but would not disappear. LCs could co-exist with globular cells and could fade after treatment. The type 2 distribution pattern was associated with older age (*p* = 0.000) and higher upper eyelid Meiboscore (*p* = 0.006). Dendritic globular cells had higher Meiboscore than Non-dendritic forms.

**Conclusions:**

Globular cells had characteristic distribution patterns and biological features different from LCs. They were associated with long-term irritation of the meibomian gland dysfunction.

## Introduction

With the help of *in vivo* confocal microscopy (IVCM), much has been learned about the microscopic environment of the cornea, nerves and cells under both physiological and pathological conditions, and new findings constantly come up (1–4).

A type of large, bright, oval-shaped cells at the sub-epithelial nerve plexus came into our sight recently. In addition to their different body shapes from other resident cells, they also had unique distribution patterns, mainly gathering in vortices and spreading along nerves in a fixed horizontal direction. These cells were not rare. We noticed their existence in some previous studies of IVCM but the authors either did not mention this special cells or mistook them for Langerhans cells (LCs) (5–7). Mastropasqua L and colleagues named them globular cells in 2006 and this name was followed by other authors (8, 9). Wolfgang J and colleagues recognized them as HLA-DR- dendritic cells (DCs) (10). However, no further information about their clinical characteristic were analyzed.

The distinction between DCs and LCs in corneal epithelium is certainly difficult and confusing. The earliest naming of dendriform immune cells (ICs) as LCs dated back to 1800s (11) and persisted for decades despite only a few studies found the presence of Birbeck granules (an exclusive marker of these resident ICs at that time) in human (11) and mouse (12) corneal epithelium. Then, Mayer et al. (13) demonstrated that only a smaller subpopulation of ICs expressed Langerin and proved to be LCs both in the central and peripheral corneal epithelium. Therefore, the naming of all ICs as LCs may be imprecise (13, 14). At present, DCs were most commonly used to identify corneal ICs in human IVCM studies (15–19). A recent review has summarized the current literature landscape to clarify the relationship between DCs and LCs, their results also in favor of that LC is a subtype of DC (14). The role of globular cells, another special type of DC, in the immune response and its relationship with LCs are still unknown. In addition, in contrast to the Well-established presence of ICs in the corneal periphery, the location of those cells in the corneal center was rarely reported (20). We reviewed the IVCM results at the corneal whorl from our clinic, summarized the distribution pattern and morphology characteristics of globular cells and analyzed their correlations with ocular surface symptoms and signs. Our observations provided new knowledge about globular cells.

## Materials and Methods

### Participants

Case records of patients from refractive and dry eye clinics at Peking University Third Hospital who underwent corneal IVCM from January 2018 to June 2020 were evaluated retrospectively. Participants with high quality corneal nerve vortex IVCM pictures were enrolled. Electronic medical records were reviewed to exclude acute ocular inflammation (infectious keratitis and conjunctivitis), ocular trauma or surgery at the time of visit and to obtain demographic variables (age and sex). Clinical variables, including tear meniscus height (TMH), tear break-up time (TBUT), meibomian gland (MG) defect and secretion, were recorded. Data on ocular surface symptoms and their severity were also collected. Among this cohort, 451 individuals (836 eyes) with a total of 1262 documents (including 426 eyes for follow-ups) of good quality IVCM images of corneal nerve vortices were enrolled in the study. The first visit IVCM images of all 836 eyes were analyzed for globular cells distribution and morphology features. Dry eye patients were treated with the same eye drops according to the common dosage regimen. Patients without LCs were assigned to the artificial tear group and treated with preservative-free artificial tears alone (0.1% sodium hyaluronate four times a day). While patients with mature LCs were assigned to the fluoromethalone group and treated with 0.1% sodium hyaluronate four times a day plus 0.1% fluoromethalone three times a day. Patients who only presented with refractive abnormalities do not require treatment. At the first 3 months, patients underwent follow-up monthly; at the next 6 months, patients were visited every 2 months; then follow-up examinations were made every 4 months. Patients could come back to the clinic at any time if they felt worse. The follow-up duration ranged from 1 month to 13 months. Clinical correlations (TMH, TBUT, MG morphology and ocular surface symptom data) of globular cells were analyzed. The construction of the database was conducted by an independent researcher who had not been involved in the care of the patients. The study was performed according to the principles of the Declaration of Helsinki and was approved by the Human Research and Ethics Committee of Peking University Third Hospital (No. M2018149). Informed consent was obtained from each of the participants.

### Clinical Variables

The central TMH of the lower eyelid, TBUT and the MG morphology of both the lower and upper eyelids were recorded by a Keratograph 5 M (OCULUS, Wetzlar, Germany). The MG morphology was further analyzed subjectively based on 1 image with optimal quality for each patient. The Meiboscore was used to grade the area of MG defect using a 4-point grading scale (0–3) as defined in a previous report (21): 0 (no dropout), 1 (<1/3 total area dropout), 2 (1/3–2/3 total area dropout), and 3 (>2/3 total area dropout). The Meibum score was obtained to grade MG secretion on a 4-point categorical scale (0–3) as reported previously (22): 0 (clear), 1 (cloudy), 2 (cloudy with debris), and 3 (inspissated, toothpaste-like).

Ten specific symptoms relating to ocular surface irritations were recorded by a questionnaire that was routinely handed out to patients of refractive and dry eye clinics at their first presentation: dryness, foreign body sensation, pain, burning, tearing, asthenopia, blurring, itching, secretions, and photophobia. Each item scores from 0 to 10 on a visual analog scale, with 0 for never and 10 for most serious. The participants were asked to grade items according to symptom severity in the latest week. Scores for each question were analyzed.

Two-dimensional pictures captured by the IVCM using a Heidelberg Retinal Tomograph 2 with Rostock Cornea Module (HRT II RCM Heidelberg Engineering Inc., Heidelberg, Germany). The device had a definition of 384 x 384 pixels over an area of 400 μm x 400 μm, with lateral spatial resolution of 0.5 μm and a depth resolution of 1–2 μm. The corneal sub-basal nerves shift centripetally and form a corneal vortex just inferior to the nose of the corneal apex (7). Corneal vortex is a land mark and plays an important role in localization because the position is fixed and easy to identify with IVCM. The central cornea over a wide of ~5.00 mm diameter (from central to peripheral area) was scanned by the same professional technician. Approximately 500 pictures were captured per eye, from the corneal epithelium to the endothelium, and at least 100 images of the sub-basal nerve plexus were obtained. Images with good quality were selected for analysis. Eyes were excluded when the corneal nerve vortex was not detected. The characteristics of globular cells and LCs distribution and morphology in the corneal vortex and peripheral area were obtained. The montages of sub-basal nerve plexus for each eye was performed with a two-dimensional graphics program (Macromedia Freehand ver. 10; Adobe Systems, San Jose, CA).

### Statistical Analysis

Continuous variables are described as the mean and standard deviation (SD) or median with interquartile range. Categorical variables are expressed as frequencies and percentages. The normality of the data distribution was verified by the Kolmogorov–Smirnov test. Continuous variables were compared with ANOVA or Kruskal-Wallis tests among three groups. Continuous data between two groups were compared using independent *t*-tests or Mann–Whitney nonparametric U-tests. Categorical variables were compared with the Chi Square test. Statistical analysis was performed using SPSS version 26.0 (IBM Corp., Armonk, New York, USA). A P value <0.05 was considered significant for all comparisons.

## Results

### Patient Demographics

A total of 836 eyes from 451 individuals (age, 60.09 ± 16.67 years; 148 males) were included. Dry eye patients without LCs (artificial tear group) were treated with 0.1% sodium hyaluronate four times a day. Patients with activated LCs (fluoromethalone group) were treated with 0.1% sodium hyaluronate four times a day plus 0.1% fluoromethalone three times a day. Among 451 individuals, 108 patients (206 eyes) had repeat visits. Their follow-up periods ranged from 1 month to 13 months. Including follow-ups, ~1262 IVCM records with good-quality images of the corneal nerve vortex were analyzed. Approximately, 595 eyes had globular cells in the corneal nerve vortex in the baseline, and 158 eyes showed changes over time.

### Morphological Features of Globular Cells in the Baseline

**Figure 1:**
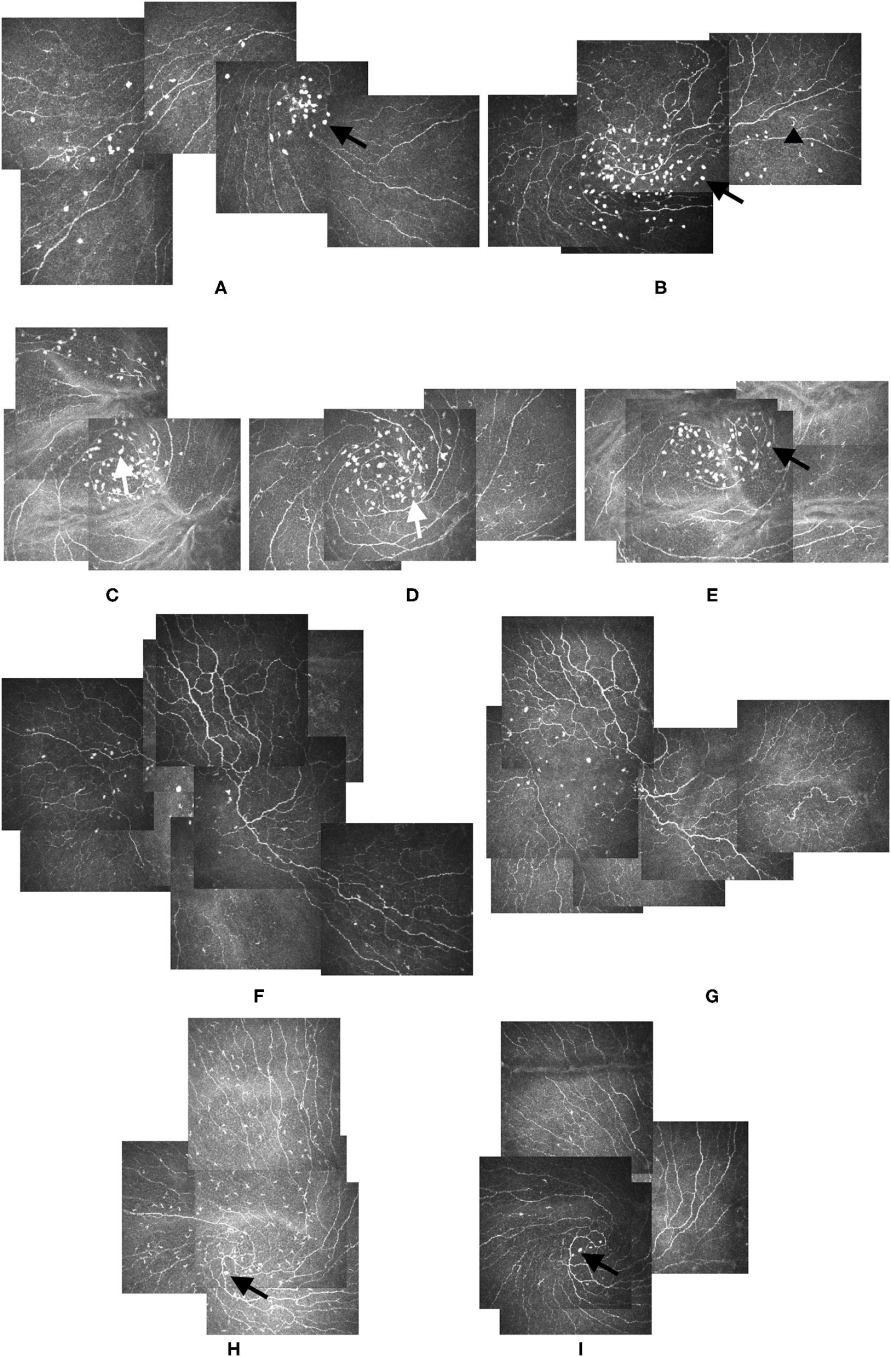
Alterations of LCs and Globular cells during follow-ups. **(A,B)** Bright oval globular cells (black arrow) could be seen in the IVCM images from study participants: 37-year-old male **(A)** and 69-year-old female **(B)**. Though of varied sizes, the globular cells were larger than the LCs (black arrowhead), and their reflectivity was higher than that of the nerves and LCs. **(C–E)** Elongated globular cells with dendrites could be identified in the images from the left eye of a 24-year-old male participant (white arrow). Images **(C–E)** were taken at baseline, 2-month, and 4-month visits, respectively. The dendrites of globular cells disappeared 4 months after the first presentation, and the globular cells became oval again (black arrow) **(E)**. **(F,G)** IVCM images from the right eye of a 77-year-old female participant showing the location and cell count of globular cells altered slightly, but the distribution pattern did not change during the 1-month follow-up **(G)**. **(H,I)** Large amounts of LCs gathered around the vortex area in the IVCM image of the left eye of a 52-year-old male participant. Globular cells could be seen at the center of the vortex (black arrow) **(H)**. Three months later, the LCs disappeared, but the globular cells remained (black arrow) **(I)**. No obvious globular cells dendrites were detected during the follow-up. LCs: Langerhans cells. IVCM: *in vivo* confocal corneal microscopy.

The globular cells had some morphological features. They were larger and brighter than LCs. Representative images of morphological characteristic of globular cells are shown in Figure 1. In most cases, the globular cells were oval cells, but in some cases, they had one or two dendrites (Figures 1A, C). The dendritic form of globular cells was different from LCs in that they had larger bodies and much shorter dendrites. The LCs were thinner and much longer (Figures 1B, C), with long processes and always accompanied corneal nerves.

### Distribution Features of Globular Cells in the Baseline

**Figure 2:**
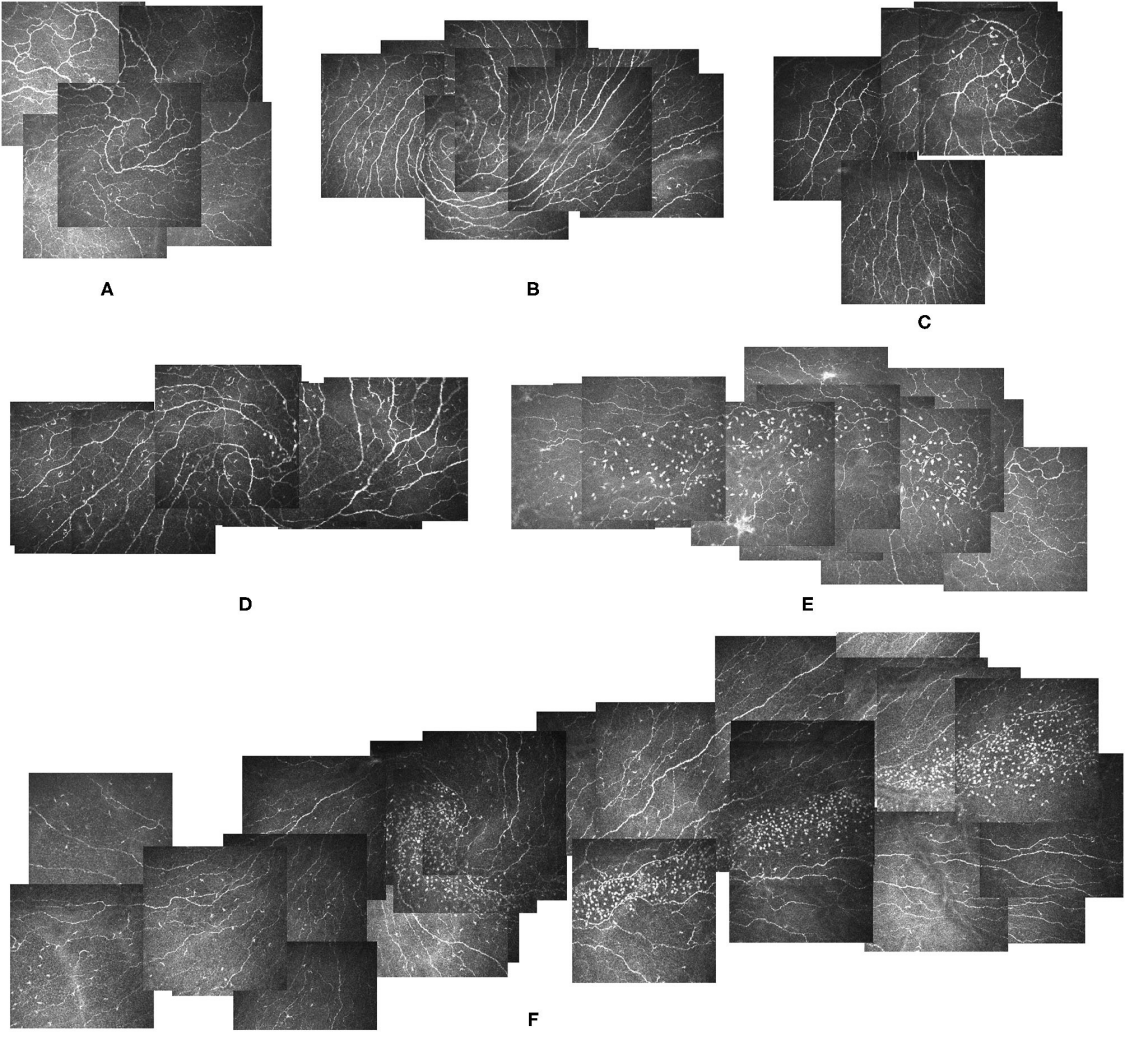
Different types of globular cells distribution patterns in the corneal vortex. **(A)** Image from a 34-year-old male participant showing a Non-globular cell pattern and a clear vortex with no globular cells. He had no LCs. **(B)** Image from a 29-year-old male participant showing a Non-globular cell pattern with LCs. **(C,D)** Images from a 36-year-old female participant showing a type 1 globular cells distribution pattern, scattered globular cells around the vortex area with LCs **(D)** and without LCs 2 months after treatment **(C)**. **(E)** Image from the right eye of a 57-year-old female participant showing a type 2 globular cells distribution pattern, with a large number of globular cells gathering within the vortex and distributing along the nerve horizontally in the temporal direction. She had no LCs in the vortex. **(F)** Image from the left eye of a 41-year-old female participant showing a type 2 globular cells distribution pattern with LCs in the vortex. The globular cells were distributed horizontally along the nerve in the temporal direction. LCs: Langerhans cells.

Three different distribution patterns of globular cells in the vortex area were detected. Type 1: some scattered globular cells (<50 cells) in the vortex area, type 2: large numbers of globular cells (≥50 cells) gathering within the vortex and distributing along the nerves to a fixed direction, mainly horizontally, either toward the nasal or temporal area, and type 3: no globular cells in the vortex. Representative images of distribution patterns of globular cells in the corneal vortex are shown in Figure 2. Approximately, 482 eyes had a type 1 distribution (57.66%, Figure 2C), and 113 eyes had a type 2 distribution (13.52%, Figure 2E). The rest had no globular cells in the vortex (28.83%, Figure 2A). The globular cells distribution patterns between a pair of eyes were not always the same. Among 384 participants with both eyes enrolled, 134 participants (34.89%) had different distribution patterns between their two eyes. LCs can be seen coexisting in the vortex in any type of globular cells distribution (Figures 2B, D, F).

The distribution of globular cells in and outside the corneal nerve vortex are shown in Table 1. Approximately 595 eyes had globular cells detected in the vortex, and 335 eyes (56.30%) also had globular cells outside the vortex. For eyes with no globular cells in the vortex (*n* = 241), 43 eyes (17.84%) had globular cells detected outside the vortex, and 198 eyes (82.16%) had no globular cell outside the vortex. Therefore, when there were globular cells in the vortex, half of them had globular cells outside the vortex, but when there were no globular cells in the vortex, they were less likely to be found outside the vortex (*p* = 0.000). Globular cells first gather at the vortex.

**Table 1:** Distribution of the globular cells in and outside the corneal nerve vortex.

**Globular cells** **outside the vortex**	**Globular cells in the vortex**	
	**Presence** **(*n* = 595)**	**Absence** **(*n* = 241)**
Presence (*n*, %)	335, 56.30%	43, 17.84%
Absence (*n*, %)	260, 43.70%	198, 82.16%

The coexisting distribution of globular cells and LCs in the corneal nerve vortex are shown in Table 2. In eyes with globular cells in the vortex (*n* = 595), 204 eyes (34.29%) had LCs coexisting in the vortex, and 391 eyes (65.71%) had no LCs in the vortex. In eyes with no globular cells in the vortex (*n* = 241), 129 eyes (53.53%) had LCs in the vortex, and 112 eyes (46.47%) had no LCs in the vortex. Fewer eyes had LCs in the vortex area when there were globular cells than when there were not (*p* = 0.000).

**Table 2:** Distribution of the globular cells and Langerhans cells in the corneal nerve vortex.

**Langerhans cells** **in the vortex**	**Globular cells in the vortex**	
	**Presence** **(*n* = 595)**	**Absence** **(*n* = 241)**
Presence (*n*, %)	204, 34.29%	129, 53.53%
Absence (*n*, %)	391, 65.71%	112, 46.47%

The distribution of LCs in and outside the corneal nerve vortex are shown in Table 3. When there were no LCs in the vortex, regardless of the presence or absence of globular cells in the vortex, nearly half of the eyes still had LCs outside the vortex (50.13% and 38.39%, respectively). However, when there were LCs in the vortex, one could always find LCs outside the vortex regardless of whether there were globular cells or no globular cells in the vortex (97.06% and 97.67%, respectively). Therefore, both LCs and globular cells could be seen in and out of the nerve vortex, but the globular cells gathered mainly within the vortex, and the LCs were mainly located outside of it.

**Table 3:** Distribution of the Langerhans cells in and outside the corneal nerve vortex.

**Langerhans cells** **outside the vortex**	**Without Langerhans cells in the vortex (*****n*** **= 503)**	
	**Globular cells Presence** **(*n* = 391)**	**Globular cells Absence** **(*n* = 112)**
Presence (*n*, %)	196, 50.13%	43, 38.39%
Absence (*n*, %)	195, 49.87%	69, 61.61%
**Langerhans cells** **outside the vortex**	**With Langerhans cells in the vortex (*****n*** **= 333)**	
	**Globular cells Presence** **(***n*** = 204)**	**Globular cells Absence** **(***n*** = 129)**
Presence (*n*, %)	198, 97.06%	126, 97.67%
Absence (*n*, %)	6, 2.94%	3, 2.33%

### Correlation of Globular Cells and Ocular Surface Status

To explore the possible ocular surface status related to vortex globular cells, the eyes enrolled for the first visited were divided into three groups according to the vortex globular cells distribution: type 1, type 2 and type 3 (Non-globular cells). Comparisons of demographics, ocular surface signs and symptoms among the three groups are shown in Table 4. The gender distribution showed no significant difference among the groups (*p* = 0.096), but the age of the type 2 group was significantly older than that of the other two groups (*p* = 0.000). The coexistence of vortex LCs was recorded, and the type 2 group had the lowest rate of LCs gathering (*p* = 0.000). TMH and Meibum scores were not significantly different among the three groups (*p* = 0.475 and *p* = 0.346, respectively), but the TBUT in type 2 group were significant shorter than type 1 group (*p* = 0.003). The Meiboscore in the upper eyelids showed a significant difference among all the groups (*p* = 0.006), and the type 2 group had a higher Meiboscore than the type 3 group (*p* = 0.045) and the type 1 group (*p* = 0.002). The type 2 group also had the highest Meiboscore in the lower eyelids, but the difference was not significant (*p* = 0.09).

**Table 4:** The demographics, ocular surface signs and symptom comparisons of the type 1 group, type 2 group, and type 3 group.

	**Type 1**	**Type 2**	**Type 3**	***p***
*n* (eyes)	482	113	241	
Age^#^	58.89 ± 16.55	68.92 ± 13.76	58.27 ± 17.01	0.000^*^
Gender (male/total, %)	91/258, 35.3%	13/62, 21.0%	44/131, 30.6%	0.096
LCs in vortex (*n*/total, %)	181/482, 37.6%	25/113, 22.1%	131/241, 54.4%	0.000^†^
**Signs**				
TMH^&^	0.19 ± 0.08	0.18 ± 0.08	0.18 ± 0.07	0.475
BUT^&^	4.08 ± 2.29	3.50 ± 1.28	3.63 ± 2.36	0.012^‡^
Upper meiboscore^&^	2 ± 2	2 ± 1	2 ± 2	0.006^||^
Lower meiboscore^&^	1 ± 1	2 ± 2	1 ± 1	0.090
Meibum score^&^	2 ± 1.75	2 ± 1.25	2 ± 2	0.346
**Symptoms**				
Dryness^&^	6 ± 4	6 ± 8	6 ± 4	0.666
Foreign body sensation^&^	4 ± 7.75	5 ± 4.5	4 ± 7	0.320
Pain^&^	1 ± 5	1 ± 8.25	3 ± 6	0.322
Burning^&^	0 ± 2.75	0 ± 4.25	0 ± 4.5	0.267
Tearing^&^	0 ± 5.75	0 ± 5.5	0 ± 6	0.909
Asthenopia^&^	7 ± 5.75	5 ± 7	6 ± 5.5	0.228
Blur^&^	2 ± 6	3.5 ± 9	1 ± 5	0.298
Itching^&^	2 ± 5	0.5 ± 8	2 ± 5	0.845
Secretions^&^	0 ± 4	0.5 ± 8	0 ± 4	0.863
Photophobia^&^	3 ± 7	5.5 ± 6.5	4 ± 7	0.344

*TMH, tear meniscus height; TBUT, tear break-up time*.

# *Mean ± standard deviation (SD)*.

& *Median ± interquartile range*.

* *ANOVA, multiple comparisons: the type 2 group was older than the type 3 (p = 0.000) and type 1 (p = 0.000) groups. The type 3 and type 1 groups were not significantly different (p = 0.662)*.

† *Chi-square: the type 1 group had more spiral LCs than the type 3 (p = 0.000) and type 2 (p = 0.000) groups. The type 1 group had more spiral LCs than the type 2 (p = 0.009) group*.

‡ *Kruskal-Wallis H, significant difference among three groups; Mann-Whitney U, pairwise comparison: the difference between the type 1 and type 2 groups was significant (p = 0.003)*.

|| *Kruskal-Wallis H, significant difference among three groups; Mann-Whitney U, pairwise comparison: the difference between the type 3 and type 2 groups was significant (p = 0.045), as was that between the type 1 and type 2 groups (p = 0.002)*.

To exclude the impact of age, case-control matching was performed between the type 2 group and the other two groups. Dryness was more severe in the type 2 group than in the type 1 group (*p* = 0.048). Additionally, the type 2 group reported more severe asthenopia than the type 1 group (*p* = 0.034). The Meiboscore showed no significant difference between the groups after case-control matching, but more eyes of the type 2 group had more than 1/3 meibomian gland dropout (Meiboscore of 2 and 3) than the other two groups (Table 5). Other clinical variables and symptoms showed no significant difference.

**Table 5:** Percentage of eyes with more than 1/3 meibomian gland dropout (Meiboscores of 2 and 3) in three groups after case-control matching.

	**Type 1 group**	**Type 2 group**	**Type 3 group**
Upper eyelid (%)	71.18%	75.00%	72.22%
Lower eyelid (%)	53.92%	59.68%	54.55%

In the type 2 group, some eyes had dendritic globular cells. To explore the possible ocular surface status related to the dendritic cell form, ocular surface signs and symptoms were compared between the eyes with dendritic globular cells (more than half of the vortex globular cells had dendrites) and Non-dendritic globular cells (more than half of the vortex globular cells had no dendrites) (Table 6). The Meiboscore of the upper eyelids in the dendritic group was higher than that in the Non-dendritic group, but the difference was not significant (*p* = 0.943). The Meiboscore in the lower eyelids was higher in the Non-dendritic group than in the dendritic group (*p* = 0.017).

**Table 6:** The demographics, ocular surface signs and symptom comparisons between dendritic and Non-dendritic globular cells.

	**Dendritic**	**Non-dendritic**	***p***
Age^#^	67.54 ± 12.45	69.04 ± 14.71	0.564
Gender (male/total, %)	4/28, 14.3%	18/57, 31.6%	0.087
**Signs**			
TMH^**#**^	0.20 ± 0.01	0.22 ± 0.14	0.132
BUT^**#**^	3.88 ± 0.18	4.02 ± 0.29	0.662
Upper meiboscore^**#**^	2.15 ± 0.97	2.14 ± 0.13	0.943
Lower meiboscore^**#**^	1.56 ± 0.13	1.96 ± 0.10	0.017^*^
Meibum score^**#**^	2 ± 1	2 ± 1	0.203
**Symptoms**			
Dryness^&^	5 ± 7.5	5 ± 6	0.482
Foreign body sensation^&^	5 ± 6	5 ± 4	0.770
Pain^&^	2 ± 9	0 ± 8	0.347
Burning^&^	0 ± 5	0 ± 3	0.479
Tearing^&^	0 ± 2.5	1 ± 3	0.064
Asthenopia^&^	5 ± 6	5 ± 7	0.770
Blur^&^	5 ± 8	4 ± 7	0.123
Itching^&^	2 ± 3	1 ± 2	0.194
Secretions^&^	1 ± 3.5	0 ± 4	0.907
Photophobia^&^	4 ± 7	5 ± 8	0.379

*TMH, tear meniscus height; TBUT, tear break-up time*.

# *mean ± standard deviation (SD)*.

& *median ± interquartile range*.

* *Independent t-tests, p < 0.05*.

### Changes of Globular Cells and LCs in Follow-Ups

Representative images of morphological characteristic of globular cells and alterations during follow-ups are shown in Figure 1. For patients who were assigned to the fluoromethalone group, the transition between the dendritic form of globular cells to the Non-dendritic form could be seen during follow-up (Figures 1C–E). Dendritic globular cells and Non-dendritic globular cells seemed to be different cell stages. During the follow-ups, the globular cells location and cell count altered slightly, but the distribution pattern did not change. The globular cells did not disappear after treatment (Figures 1F–I). In contrast to globular cells, dramatic changes in LCs could be seen in the follow-up. The LCs disappeared after treatment in some cases, but they were not replaced by globular cells (Figures 1H,I). In some cases, LCs increased in the follow-ups, but there was no globular cells accumulation before LCs increased. Therefore, the LCs and globular cells would not interchange.

## Discussion

The cornea is a relatively immunologically privileged site because it lacks lymphatic circulations (23). However, the cornea is susceptible to various inflammatory diseases mediated by immunity, such as infections and autoimmunity, which are attributed to resident myeloid cell populations, such as epithelial LCs and stromal macrophages and DCs, which can function as antigen-presenting cells (APCs) (23, 24). The distribution, density and morphology of those cells are closely related to the physiological and pathological state of the cornea (23–26). Until now, there have been only a few reports about the location of LCs or DCs in the corneal center in contrast to the Well-established presence of APCs in the corneal periphery (20). The origin and migration of DCs are still unknown.

In the present study, we analyzed the globular cells, a subset of DCs (9, 17). The globular cells had specific morphological and distribution features which differed from the LCs (another subtype of DCs). The globular cells were oval shape and relatively larger and brighter than LCs. In some cases, the globular cells were elongated with short dendrites, but their cell bodies were not as thin as LCs, and their dendrites were not that long either. LCs never showed round or oval shape, even in the immature stage still showing processes accompanied corneal nerves (23, 27). The globular cells were found mainly gathering in the corneal vortex, and when in large amounts, the globular cells would distribute horizontally along the nerves, either to the temporal or nasal direction. Furthermore, when no globular cells was detected in the vortex, it was less likely to find globular cells outside the vortex. It seemed that the globular cells were more congregated in the central cornea and decreased rapidly toward the periphery, indicating that globular cells might migrate from the vortex to the peripheral cornea. The distribution of LCs was completely different. According to our research, if LCs were located outside the vortex, one may not find LCs in the vortex. However, if LCs were detected in the vortex, one can always find LCs outside the vortex. These results were consistent with previous studies showing that most LCs resided in the corneal periphery, with LCs numbers decreasing rapidly toward the corneal center under Non-pathological conditions (27). In pathological terms, immature LCs, which might still be motile, were able to transform into active corneal immune responses with dendrite-like processes under appropriate stimuli (28, 29). LCs were reported to migrate centripetally from the periphery and limbus toward the central cornea during maturation (20).

The best way to confirm globular cells origin is immunohistochemical analysis, but this was infeasible *in vivo*. Therefore, in our present study we could only speculate the origin of globular cells. Previous study suggest that globular cells were HLA-DR-, but the data were not shown and no further reports could be found (10). The DC precursors exist in the blood and display many different properties (30). High numbers of CD14+ (a precursor-type cell surface marker associated with undifferentiated DCs) cells were demonstrated mainly in mouse central corneal stroma and were negative for MHC class II (31). The anterior stroma has a mixed population of DCs and lymphocytes (23). A pronounced accumulation of MHC class II+ DCs were found in the limbal and peripheral stroma, but the center, however, contained exclusively MHC class II- DCs both in the murine (31) and human corneas (13). Macrophages are another APC in the stroma, but they populate only in the posterior stroma peripheral and paracentral both in mice and the human cornea (13, 31, 32). Large numbers of DCs in the cornea remain in an undifferentiated state and could be skewed toward either DC- or macrophage-like profiles in response to different factors (32). Then, the globular cells in our study possibly be DCs in the anterior stroma and migrate anteriorly to the epithelium. Further immunohistochemical researches are needed to confirm our hypothesis.

The characteristic distribution pattern of the globular cells along the nerves in one fixed horizontal direction made us consider the possibility of cell transport along or with corneal nerves. A recent study confirmed our suspicion, during a 44-day observation period, these globular cells migrated closely followed corneal nerve migration pattern in a healthy 35-year-old female (33). And this case also verified that globular cells mainly gathered in the corneal vortex. However, this study provided a possible migration in the same sub-basal nerve plexus level. The transport of globular cells at different corneal layers remains unknown. A weak relationship was found between epithelial cells and corneal nerve movement, and their rates of migration were comparable; therefore, they were assumed to migrate together as a unit (34). A so-called X, Y and Z hypothesis also suggests a way of anterior migration of epithelial cells (35). Moreover, LCs have been found to be contiguous to nerves of the basal epithelial plexus in histopathologic specimens (34). The authors speculated that the LCs in the central corneal epithelium might come from the anterior stroma and migrate anteriorly along the nerve toward the inferocentral cornea. They may take advantage of the potential space around the nerve as a path of least resistance to movement through the basal epithelium (34). The globular cells might also migrate with or along the corneal nerves through this potential space. If so, the nerve structure of the vortex and its connection with the nerves beneath needs to be analyzed closely since the vortex may play a hub role in cell transportation from the anterior stroma to the epithelium. It was reported that the stromal nerve bundles entered the anterior cornea at the limbus, coursing centrally and anteriorly (34, 36). As stromal nerves reach the anterior stroma near the interface between the stroma and Bowman's layer, the nerves form a profusely branched network called the subepithelial nerve plexus (36). Some studies also presented the possible invasion of nerves to central corneal areas from the underlying structures of the cornea. All these findings were in line with the assumption that globular cells might be DCs in the stroma that use nerve structures to move anteriorly to the subepithelial nerve plexus under certain stimuli.

Much is known about LC function in corneal and ocular surface disease. An earlier set of studies suggested that LCs in most tissues were geared toward maintaining homeostasis (23) and had a pivotal role in regulating ocular surface immune processes (29). Resident LCs patrol the tissues, sampling self-antigen while on the watch for foreign antigen, maintaining tolerance and inducing immunity (23, 37, 38). Aside from LC density, activated LCs are important for LC function and immuno-inflammatory status (39, 40). LCs and corneal nerves may have close communication and could reciprocally affect each other's function for maintaining ocular surface homeostasis. It has been reported that the loss of LCs in the corneal epithelium resulted in significant downregulation of neurotrophic factors, loss of corneal nerves and aggravated corneal erosion (26). Additionally, corneal innervation affects the activation status of LCs under normal and pathological conditions (41). LCs in the skin were shown to be closely related to cutaneous nerves while targeting them with secretion of nerve products (41, 42). Neurotrophic factors and neurotransmitters also regulate LC function (43). The globular cells morphology characteristic and location with respect to nerves suggested an active neuro-immunological crosstalk and may participant in immune surveillance and ocular surface homeostasis maintenance. We could not clarify the role of globular cells here, but according to our study, the globular cells had very different biological features from LCs; thus, globular cells may perform different functions from LCs. As Smedowski et al., found round cells ratio and shape revealed differences in cytological features of infectious keratitis (44). In the present study, globular cells possibly be infiltrating leukocytes that are related to micro-erosions developing as a long result of dry eye disease, especially accompanied with meibomian gland dysfunction (MGD).

In our research, the globular cells may change their locations, but their distribution patterns would not change in the follow-ups and they would not disappear after eye drop treatment. One possible explanation for this finding might be attributed to an external incentive of globular cells gathering, which was persistent and difficult to remove by regular treatment such as artificial tears or fluorometholone. Our results indicated that compared with the other two groups, participants with type 2 distribution had the most severe MGD (higher Meiboscore) and the shortest TBUTs. MGD could not easily recover and may be a persistent trigger for globular cells. However, in addition to MGD, type 2 participants were also the oldest group. To exclude the possible impact of age, we made a case-control match among the groups, and the results showed that the type 2 group still had the most cases of severe MGD (Meiboscores of 2 and 3), but the difference was not significant. MGD was previously reported to be associated with age (45). We could not differentiate the concurrent influences of age and MGD on globular cells distribution in our research, but the result that globular cells tended to gather in large amounts in older patients with more severe MGD suggests that long-term MGD might be a cause for globular cells gathering.

Globular cells with dendrites were identified in some cases. Dendritic globular cells and Non-dendritic globular cells may be two cell activating statuses. We compared their ocular surface index and found that dendritic globular cells had more severe MGD in upper eyelids than Non-dendritic globular cells. No significant difference was found in age between the two groups. MGD might also influence the cell status of globular cells.

All the results showed a possible relationship between MGD and globular cells in terms of cell gathering and dendrite transformation. The characteristic horizontal distribution of globular cells along the nerves in type 2 also indicated that there might be persistent irritation from the MG to the interpalpebral zone, such as orifice rubbing. The grade of Meibum showed no significant correlation with globular cells; however, further analysis of the Meibum component in future studies might provide new information.

Our research had some limitations. Immunohistochemical staining may be the best way to identify globular cells but it is not feasible *in vivo*. The relationship between the immunohistochemical staining of the corneal DCs *in vitro* and their image under IVCM *in vivo* remains unclear. In this study, we could only speculate the origin and migration of globular cells according to their characteristics observed by IVCM. New technologies are needed to confirm our results. Additionally, the IVCM device used in study is outdated. However, our observations still have some clinical implications. We hoped to draw attention from peer researchers to the existence of this special type of DCs. Their relationship with MGD provided new pathological information for ocular surface diseases. Also, their unique distribution pattern along the corneal nerves are puzzled and needed to dig further. The functional relationship of globular cells and corneal nerves at the molecular level needs to be further analyzed.

In summary, the globular cells, a type of bright and oval DCs, were identified in the corneal vortex with a horizontal distribution along nerves. They were different from LCs in terms of cell morphology and biological behaviors. They could transform between dendritic and Non-dendritic forms. Their gathering and transformation were related to MGD and old age.

## Data Availability Statement

The raw data supporting the conclusions of this article will be made available by the authors, without undue reservation.

## Ethics Statement

The studies involving human participants were reviewed and approved by Human Research and Ethics Committee of Peking University Third Hospital (No. M2018149). The patients/participants provided their written informed consent to participate in this study.

## Author Contributions

RH, ZL, and YC setup the protocol and recruited the participants. RH collected and analyzed the data, created the figures, and contributed to the writing. ZL and YC discussed the data and participated in writing manuscript. CH, DJ, HW, and SG provided statistical advice and oversaw the statistical analysis. XL setup the protocol and oversaw the final manuscript. All authors contributed to the article and approved the submitted version.

## Funding

This work was supported by the National Natural Science Foundation of China (grant number: 81900826).

## Conflict of Interest

The authors declare that the research was conducted in the absence of any commercial or financial relationships that could be construed as a potential conflict of interest.

## Publisher's Note

All claims expressed in this article are solely those of the authors and do not necessarily represent those of their affiliated organizations, or those of the publisher, the editors and the reviewers. Any product that may be evaluated in this article, or claim that may be made by its manufacturer, is not guaranteed or endorsed by the publisher.
